# Draft genome sequences of nine marine-derived *Dothideomycetes* strains from Sugashima, Japan

**DOI:** 10.1128/mra.01318-25

**Published:** 2026-03-10

**Authors:** Kyoka A. Adachi, Yuri Fujita, Gohta Goshima, Gakuho Kurita

**Affiliations:** 1 Sugashima Marine Biological Laboratory, Graduate School of Science, Nagoya University 89301, Toba, Japan; 2 Graduate School of Environmental, Life, Natural Science and Technology, Okayama University, Okayama, Japan; University of Maryland School of Medicine, Baltimore, Maryland, USA

**Keywords:** *Dothideomycetes*, black yeast

## Abstract

Marine-derived fungal species belonging to the class *Dothideomycetes* show remarkable variability in their life forms, such as obligate multicellularity and facultative multicellularity. The genetic basis of this diversity remains unknown. Here, we report the draft genome sequences of nine *Dothideomycetes* strains, including the genera *Cladosporium*, *Neophaeotheca*, *Zalaria*, and *Neodevriesia*, ranging from 19 to 36 Mb in size.

## ANNOUNCEMENT

*Dothideomycetes* are a diverse class within *Ascomycota*, inhabiting various niches, including marine environments (1). Several species, such as *Hortaea werneckii,* show facultative multicellularity, alternating their mode of growth/division depending on cell density or nutrient availability, whereas others include obligately multicellular filamentous fungi (e.g., *Cladosporium*) (2–5). Despite these cell-biologically intriguing traits, genomic resources for *Dothideomycetes* remain limited. In this study, nine *Dothideomycetes* strains were isolated from the marine environment at the Sugashima Marine Biological Laboratory, Nagoya University, on Sugashima Island, Toba, Japan (34.49°N, 136.88°E). Marine organisms and seawater from near-shore and an outdoor tank (see Fig. 1 of [3]) were plated onto YPD or EMM plates containing antibiotics (20 µg/mL carbenicillin, 100 µg/mL chloramphenicol, and 10 µg/mL tetracycline) and incubated at 25°C (3). DNA was extracted from cloned colonies boiled in 0.25% SDS for 5 min. Barcode sequencing of the ITS (primers: ITS1 [TCCGTAGGTGAACCTGCGG] and ITS4 [TCCTCCGCTTATTGATATGC]) and the LSU D1/D2 region (NL1 [GCATATCAATAAGCGGAGGAAAAG] and NL4 [GGTCCGTGTTTCAAGACGG]) enabled identification at the species or genus level for strains NU13, NU230, NU234, NU284, and NU419 based on the best BLAST matches to type materials. The phylogenetic positions of NU197, NU332, NU457, and NU459 could not be resolved. These nine strains were chosen for whole-genome sequencing.

**Fig 1:**
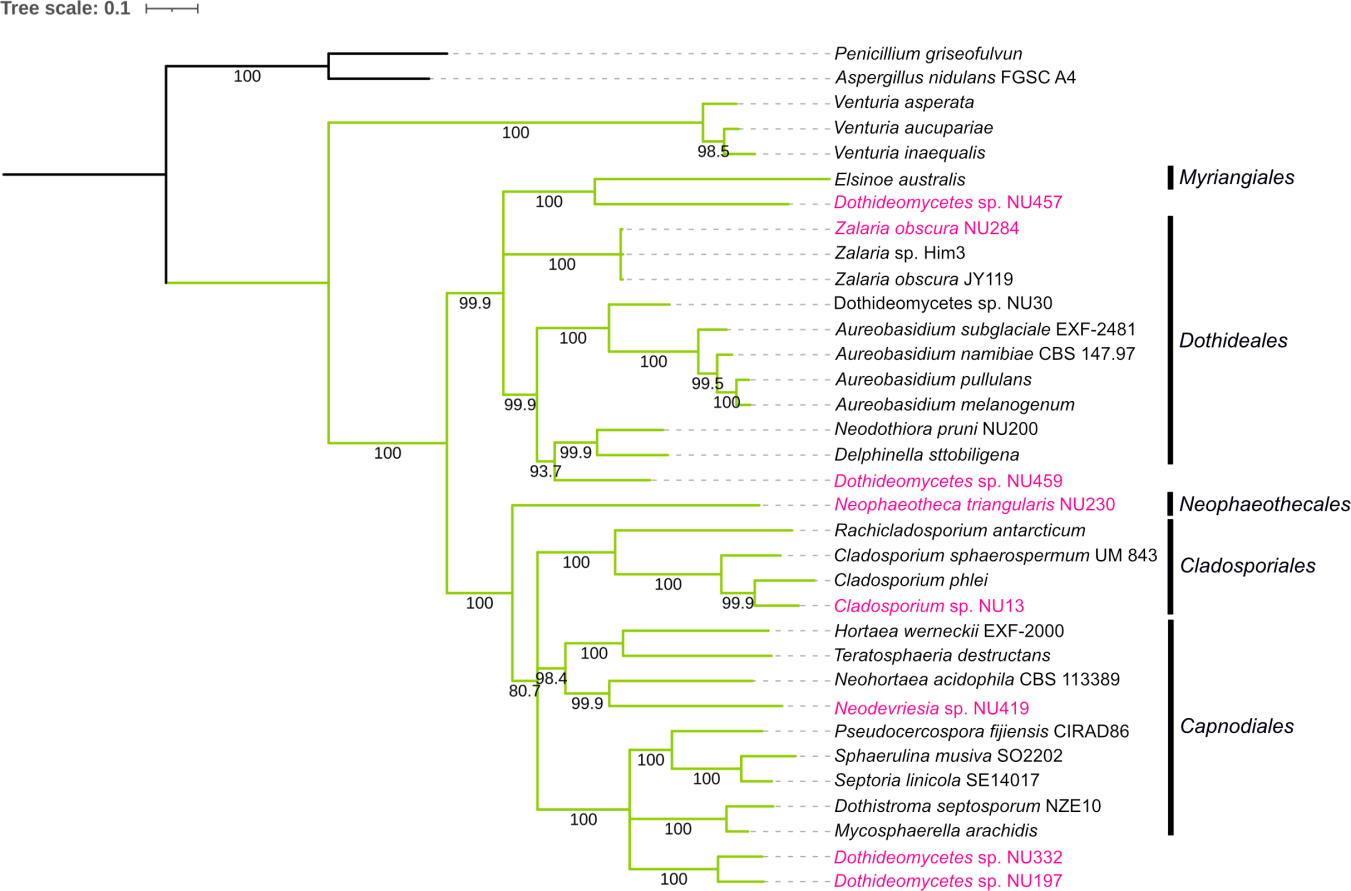
Phylogenetic relationships within the class *Dothideomycetes*. Phylogenetic reconstruction was performed using the maximum-likelihood (ML) method based on concatenated amino acid sequences of DNA-directed RNA polymerase II core subunit (RPB2), α-tubulin, eukaryotic translation initiation factor 5 (TIF5), and DNA topoisomerase I (TOP1). These protein sequences were obtained from single-copy orthologs identified in the BUSCO output (6). Multiple sequence alignment was generated using MAFFT v7.520 (7) and trimmed with ClipKIT v1.4.1 (8) using the “kpi” option. The aligned sequences were manually concatenated. ML-based tree inference was conducted with IQ-TREE v2.1.4 (9) using the option “-m MFP -bb 1000 -alrt 10.” Branch labels represent bootstrap values. *Penicillium griseofulvum* and *Aspergillus nidulans* FGSC A4 were used as outgroups. Green: *Dothideomycetes*; Magenta: strains sequenced in this study.

Genomic DNA was extracted from NU13, NU230, and NU234 cells grown exponentially in PDB or YPD liquid medium using a Plant Mini Kit (Qiagen). For the others, DNA was extracted from cells grown exponentially in YPD liquid medium using a Dr. GenTLE kit (Takara Bio). DNA samples were examined by electrophoresis, and concentration and purity were assessed using a NanoDrop 2000c spectrophotometer. Library preparation and sequencing were conducted by Novogene or BGI Genomics. Libraries were prepared using the VAHTS Universal Plus DNA Library Prep Kit (Vazyme) for NU13, NU284, and NU419; a whole-genome enzyme digestion-based method (Yeasen) for NU197 and NU332; and the NEBNext Ultra II DNA Library Prep Kit (NEB) for NU230, NU234, NU457, and NU459. All sequences were generated as 2× 150-nucleotide paired-end reads. Read quality was checked using FastQC v0.11.9 (10) and fastp v0.23.2 (11). *De novo* assemblies generated using MaSuRCa v3.3.0 (12) or SPAdes v3.13.1 (13) were polished with Pilon v1.24 (14) to correct assembly errors. Genome sizes ranged from 19 to 36 Mb (Table 1). Ploidy estimation with ploidyNGS v3.1.2 (15) suggested NU234 and NU284 were diploid, whereas the other strains were haploid. Genome completeness was assessed using Benchmarking Universal Single-copy Orthologs (BUSCO) v5.7.0 (6) with the dothideomycetes_odb10 data set. All strains showed >90% completeness.

**TABLE 1:** Summary of genome sequencing performed in this study

Strain	Isolation date	Site	Source	SRA accession no.	Draft genome accession no.	Species (based on ITS and NL barcode sequences)	Ploidy estimation	No. of contigs	Genome size (bp)	N50 (bp)	Sequencing depth	G+C content (%)	Sequencer	Assembler
NU13	March 2020	Outdoor tank	Seaweed	DRR724774	BAAIJQ000000000	*Cladosporium* sp.	Haploid	191	32,226,018	841,021	154.6	52.8	NovaSeq X	SPAdes v3.13.1
NU197	October 2021	Outdoor tank	Seawater	DRR724775	BAAIJR000000000	*Dothideomycetes* sp.	Haploid	335	35,723,521	294,122	270.6	52.5	DNBSEQ-G400	SPAdes v3.13.1
NU230	November 2021	Seafloor	Soft coral	DRR728498	BAAIJX000000000	*Neophaeotheca triangularis*	Haploid	136	25,422,514	389,357	188.8	53.7	NovaSeq X	SPAdes v3.13.1
NU234	November 2021	Seafloor	Sponge	DRR728499	BAAIJY010000000	*Neophaeotheca triangularis*	Diploid	6,705	31,436,815	8,013	146.3	53.7	NovaSeq X	MaSuRCa v4.1.0
NU284	April 2022	Seafloor	Sponge	DRR724776	BAAIJS010000000	*Zalaria obscura*	Diploid	2,925	30,100,000	17,358	145.3	53.5	NovaSeq X	MaSuRCa v3.3.0
NU332	October 2022	Seafloor	Sponge	DRR724773	BAAIJT010000000	*Dothideomycetes* sp.	Haploid	81	30,397,331	902,196	337	53.4	DNBSEQ-G400	SPAdes v3.13.1
NU419	October 2023	Outdoor tank	Starfish	DRR724777	BAAIJU010000000	*Neodevriesia* sp.	Haploid	242	26,825,905	680,676	261.7	58.9	NovaSeq X	SPAdes v3.13.1
NU457	January 2024	Pier	Seawater	DRR724778	BAAIJV010000000	*Dothideomycetes* sp.	Haploid	64	18,995,028	708,280	265.4	51.9	NovaSeq X	SPAdes v3.13.1
NU459	February 2024	Outdoor tank	Seawater	DRR724779	BAAIJW010000000	*Dothideomycetes* sp.	Haploid	166	24,513,454	302,953	205.3	54.0	NovaSeq X	SPAdes v3.13.1

Concatenated amino acid sequences of RPB2, α-tubulin, TIF5, and TOP1, in the BUSCO output, were used to construct a phylogenetic tree, which placed the eight strains within the class *Dothideomycetes* (Fig. 1). The NU234 strain, identified as *Neophaeotheca triangularis* based on barcode sequencing, did not yield the sequences necessary for inclusion in the phylogenetic tree.

Diverse cell division patterns exist within *Dothideomycetes* (2, 3). The draft genome sequences presented here enrich genomic resources, providing a foundation for deeper understanding of their biology.

## Data Availability

Raw sequencing reads were deposited in the DNA Data Bank of Japan (DDBJ) under BioProject accession number PRJDB35956. Accession numbers for BioSample, DRA, and draft genomes for each strain are listed in Table 1 (sequences are available via DDBJ getentry: https://getentry.ddbj.nig.ac.jp/top-e.html).
